# Plasma miR-153 and miR-223 Levels as Potential Biomarkers in Parkinson’s Disease

**DOI:** 10.3389/fnins.2022.865139

**Published:** 2022-05-17

**Authors:** Li Wu, Qian Xu, Mengxi Zhou, Yajing Chen, Chunyan Jiang, Yuhan Jiang, Yin Lin, Qing He, Lei Zhao, Yourong Dong, Jianren Liu, Wei Chen

**Affiliations:** Department of Neurology, Shanghai Ninth People’s Hospital, Shanghai Jiao Tong University School of Medicine, Shanghai, China

**Keywords:** Parkinson’s disease, α-synuclein, plasma, miRNAs, rapid eye movement sleep behavior disorder

## Abstract

**Background:**

Small molecule RNAs (miRNAs) could induce downregulation of α-synuclein (SNCA) expression by binding the 3’ untranslated region of SNCA, thus playing an important role in the pathogenesis of Parkinson’s disease (PD). Recent studies suggest that SNCA-related miRNAs in saliva are promising PD biomarkers. Research on those miRNAs in plasma is rare in patients with PD.

**Objective:**

To detect the plasma expression levels of three SNCA related miRNAs (miR-7, miR-153, and miR-223) in PD, and to explore their diagnostic value and associations with clinical phenotype.

**Methods:**

MiR-7, miR-153, and miR-223 levels were detected in the plasma of 75 PD patients and 73 normal controls (NCs) *via* real-time quantitative polymerase chain reaction. The receiver operating characteristic (ROC) curves were delineated to evaluate their diagnostic value in PD. In addition, their associations with demographic, key motor, and non-motor symptoms were explored by serial scales.

**Results:**

The expression levels of plasma miR-153 and miR-223 were significantly decreased in patients with PD relative to NCs. The area under the ROC curve separating PD from NCs was 63.1% for miR-153 and 86.2% for miR-223, respectively. The plasma miR-153 level in *de novo* PD was lower than that in treated patients (*p* = 0.006), its level increased gradually with disease duration (*r* = 0.358, *p* = 0.002) and Unified Parkinson’s Disease Rating Scale Part III score (*r* = 0.264, *p* = 0.022). Plasma miR-223 level was decreased in patients with clinical possible rapid eye movement sleep behavior disorder (cpRBD) compared with those without cpRBD (*p* < 0.001), and its level was negatively associated with RBDSQ score (*r* = -0.334, *p* = 0.003). Multiple linear regression analysis revealed that disease duration (*p* = 0.049) was the independently associated factor of miR-153 level; whereas, RBDSQ (*p* = 0.009) was related to miR-223 level in PD.

**Conclusion:**

Plasma miR-153 and miR-223 levels could be potential biomarkers of PD.

## Introduction

Parkinson’s disease (PD) is a common neurodegenerative disease that usually affects the elderly (Ascherio and Schwarzschild, 2016; Poewe et al., 2017) and brings a heavy economic burden to the patients’ families and society. Pathologically, it is characterized by the selective loss of dopaminergic neurons in the substantia nigra and Lewy bodies in the remaining neurons (Homayoun, 2018). Currently, the clinical diagnosis of PD is mainly based on clinical examination and neuroimaging evaluation (Postuma et al., 2015b). Misdiagnosis and missed diagnosis often occur in clinical practice. Therefore, the identification of early objective biomarkers is one of the main priorities in PD research.

As one kind of endogenous non-coding RNA molecules, small molecule RNAs (miRNAs) can inhibit protein-coding genes by affecting mRNA translation and circulation in the blood and cerebrospinal fluid (CSF) and are involved in the pathogenesis of many diseases, including PD (Ramaswamy et al., 2018; Goh et al., 2019; Van den Berg et al., 2020). The ideal biofluid sample is CSF because of its close interaction with the pathological brain. However, CSF can only be obtained through a lumbar puncture, which cannot be well accepted by the patients (Starhof et al., 2019). Blood is relatively easy to access. Blood cells could partly reflect the physiological and pathological stimuli and treatments because of their specific cellular organizations and connections with most of the tissues in the body including the brain (Soreq et al., 2014). Intriguingly, it has been reported that the transcriptomic patterns of brain and peripheral blood overlap by approximately 81.9% (Liew et al., 2006). Therefore, assessment of miRNAs in blood could be one practical strategy to identify biomarkers in PD (Arshad et al., 2017).

The importance of α-synuclein (SNCA) in PD is seminal. Mutations in the SNCA gene were the first reported genetic types in familiar PD (Polymeropoulos et al., 1997). Moreover, SNCA is the major component of Lewy bodies in sporadic PD and it has been demonstrated that the expression levels of SNCA are increased in sporadic patients with PD, as well as in animal models with PD (Spillantini et al., 1998; Vila et al., 2000). Interestingly, higher levels of normal non-mutated SNCA could also induce SNCA aggregation and Lewy body formation in PD, suggesting that regardless of the mutational status of the SNCA gene, even small changes in SNCA levels could trigger PD development and progression (Singleton et al., 2003). Therefore, the expression levels of SNCA may play an important role in triggering PD progression and could be potential biomarkers for PD.

Several miRNAs were reported to be able to regulate SNCA gene expression, and the specific ones are miR-7, miR-153, and miR-223, as predicted by three miRNA target gene prediction software programs (TargetScan Human 7.1, MicroCosm Targets)^1^. A recent study reported that two SNCA-related miRNAs, miR-153 and miR-223 levels, were decreased in the saliva of patients with PD, and could serve as promising PD biomarkers (Cressatti et al., 2020). Whereas their corresponding expression levels in the blood of patients with PD were elusive. In this study, we detected the expression levels of three SNCA related miRNAs (miR-7, miR-153, and miR-223) in plasma and explored their diagnostic value and associations with the clinical phenotype in PD.

## Materials and Methods

### Subjects

This study was approved by the Medical Ethics Committee of Shanghai Ninth People’s Hospital, Shanghai Jiao Tong University School of Medicine, Shanghai, China. All the participants provided written informed consent. From September 2019 to September 2021, seventy-five patients with PD and 73 normal controls (NCs) were included in this study. Patients with PD were examined and diagnosed by at least two experienced neurologists from the Department of Neurology of our hospital, according to the Movement Disorder Society (MDS) clinical diagnostic criteria of PD (Postuma et al., 2015a,b). NCs were recruited from the health examination center of our hospital, and they had no obvious neurological disorders such as stroke, brain tumor, epilepsy, parkinsonism, related disorders, etc. Both the participants in the PD and NC groups were matched for age, gender, and Chinese version of the Mini-mental State Examination (MMSE) score.

### Total miRNA Extraction From Plasma

In total, two milliliters of peripheral blood were drawn from the cubital vein into a vacuum blood tube with ethylenediaminetetraacetic acid (EDTA) and then the plasma was aliquoted (200 μl) into a sterile tube and stored at −80°C. Plasma samples of participants were collected to extract plasma miRNAs. Total miRNA was extracted and purified from plasma samples using the miRNeasy Serum/Plasma Kit (Qiagen, Hilden, Germany, Catalog number 217184). All the operations were conducted in strict accordance with the manufacturer’s instructions. The synthetic *Caenorhabditis elegans* miR-39 (cel-miR-39, Qiagen; 219610) was added to plasma after lysis as an external reference, which is devoid of sequence homology to human miRNAs.

### Reverse Transcription and Real-Time Quantitative Polymerase Chain Reaction Experiment

RNA was reverse transcribed with a miScript reverse transcription kit (Qiagen, Catalog number 218161). The expression of miRNAs was calculated by real-time quantitative polymerase chain reaction (RT-qPCR) using the miScript SYBR Green PCR kit (Qiagen, Catalog number 218073). The American ABI7500 Fluorescent Quantitative PCR instrument was used for detection. Specific miRNA primers were obtained from TIANGEN Biotech Co., Ltd. (Beijing, China). The calculation of the relative miRNA expression levels was analyzed using the delta–delta cycle threshold value (2^–ΔΔ*Ct*^) method. The expression levels of different miRNAs were normalized with cel-miR-39 and compared as described previously (Schmittgen and Livak, 2008). The expression levels of miR-7, miR-153, and miR-223 in patients with PD and NCs were compared.

### Clinical Evaluation

For the patients with PD, motor severity was measured with a modified Hoehn and Yahr (H&Y) stage (Hoehn and Yahr, 1967) and Unified Parkinson’s Disease Rating Scale part III (UPDRS-III) (Richards et al., 1994). clinical possible rapid eye movement sleep behavior disorder (cpRBD) was screened by using REM Behavior Disorder Screening Questionnaire (RBDSQ) (Nomura et al., 2011). Freezing of gait (FOG) was considered present when subjects had more than one score on item 3 of the Freezing of Gait questionnaire (FOG-Q) (Giladi et al., 2000). We used MMSE (Katzman et al., 1988) and the Chinese Version of the Montreal Cognitive Assessment-Basic (MoCA-BC) (Xu et al., 2021) to measure patients’ cognitive function. Autonomic dysfunction was assessed by the Scales for Outcomes in PD Autonomic Dysfunction (SCOPA-AUT) (Verbaan et al., 2007). Olfactory function was assessed by SS-16 as in the previous report (Chen et al., 2012). The severity of depressive symptoms was assessed by the 17-item Hamilton Rating Scale for Depression (HAMD-17), and a score of ≥8 was regarded as depression (Hamilton, 1960). Newly diagnosed, drug naïve patients were regarded as *de novo* PD. For medicated patients, levodopa equivalent daily dose (LEDD) was calculated.

### Statistical Analysis

SPSS version 23.0 (IBM Corporation, Armonk, NY, United States) was used for statistical analysis. Continuous variables are expressed as the means ± SD or medians [interquartile ranges (IQRs), Q1–Q3]; categorical variables are expressed as frequencies and percentages. Comparisons of means between the two groups were performed using the independent *t*-test or the Mann–Whitney *U* test as appropriate. Since the distribution of miRNA expression levels in each group was highly skewed, the expression level was log-transformed for normal distribution. The chi-square or Fisher exact test was used for comparing proportions. The diagnostic value of each miRNA was evaluated by delineating the receiver operating characteristic (ROC) curve and selecting the point with the maximum Yuden index as the cut-off value and the area under the curve (AUC) was calculated accordingly. In addition, the differences between the three miRNAs in PD subtypes (*de novo* PD vs. medicated PD, cpRBD positive vs. cpRBD negative, etc.) were also compared. We analyzed the continuous variables by one-way ANOVA or non-parametric Kruskal–Wallis tests, depending upon whether the data were normally distributed or not. The *p*-values for these three group comparisons were adjusted using the Bonferroni method. Correlations between the three miRNA levels and clinical data (disease duration, H&Y stage, UPDRS-III, RBDSQ, etc.) in PD were assessed using the Pearson’s correlation coefficient. Multiple linear regression analysis was used to explore the independently associated factors of mRNA levels in PD. The test level (α) was 0.05.

## Results

### General Characteristics

The general characteristics of patients with PD and NCs were shown in Table 1. Age, gender, and MMSE score were consistent between patients with PD and NCs. For patients with PD, the median disease duration was 2.0 years, and 34.7% (*n* = 26) of the patients were *de novo* patients with PD. In total, eighteen patients (24.0%) had a family history of PD or tremors. In total, forty-six patients (61.3%) had H&Y stage ≥2. The frequency of hyposmia and cpRBD were 66.7 and 49.3%, respectively. Nine patients (12.0%) had wearing-off and two patients (2.7%) had dyskinesia. None of the patients received deep brain stimulation.

**TABLE 1:** General characteristics in patients with PD and NCs.

Items	PD (*n* = 75)	NC (*n* = 73)	*t*/*z*	*p*-value
**Demographic data**				
Age (years)	68.0 (62.0, 72.0)	67.0 (64.0, 70.5)	–0.221	0.825
Male, *n* (%)	44 (58.7%)	33 (45.2%)	2.686	0.101
Disease duration (years)	2.0 (1.0, 5.0)	NA		
Family history, *n* (%)	18 (24.0%)	NA		
*de novo* PD, *n* (%)	26 (34.7%)	NA		
**Motor features**		NA		
H&Y stage ≥2 (%), *n* (%)	46 (61.3%)			
UPDRS-III score	19.0 (14.0, 30.0)			
Motor subtype, *n* (%)				
Tremor-dominant type (TD)	24 (32.0%)			
Akinetic-rigid type (A-R)	41 (54.7%)			
Mixed type	10 (13.3%)			
Side most affected, *n* (%)				
Left	27 (36.0%)			
Right	29 (38.7%)			
Bilateral	19 (25.3%)			
FOG-Q	3.0 (1.0, 8.3)			
FOG+, *n* (%)	23 (30.7%)			
**Non-motor features**				
NMSQ	8.0 (4.0, 12.0)	NA		
SS-16	7.0 (6.0, 9.0)	NA		
Hyposmia, *n* (%)	50 (66.7%)	NA		
RBDSQ	5.0 (1.0, 8.0)	NA		
cpRBD, *n* (%)	37 (49.3%)	NA		
HAMD-17	5.0 (2.0, 9.0)	NA		
Depression, *n* (%)	25 (33.3%)	NA		
SCOPA-AUT	13.0 (9.0, 18.0)	NA		
MMSE	28.00 (25.00, 29.00)	28.00 (25.00, 30.00)	–0.730	0.465
Motor complications				
Wearing-off, *n* (%)	9 (12.0%)			
Dyskinesia, *n* (%)	2 (2.7%)			
Plasma miRNAs level				
miR-7	0.89 (0.47, 1.66)	1.00 (0.33, 2.43)	–0.604	0.546
miR-153	0.68 (0.30, 1.10)	1.00 (0.48, 2.20)	–2.757	0.006*
miR-223	0.18 (0.05, 0.37)	1.00 (0.41, 2.28)	–7.630	< 0.001**

PD, Parkinson’s disease; NC, normal controls; H&Y stage, Hoehn and Yahr stage; UPDRS-III, Unified Parkinson’s Disease Rating Scale part III; TD, tremor dominant; A-R, akinetic-rigid; FOG-Q, Freezing of Gait Questionnaire; FOG, freezing of gait; +, positive; NMSQ, Non-motor Symptoms Questionnaire; SS-16, 16-item odor identification test from Sniffin’ Sticks; RBDSQ, Rapid Eye Movement Behavior Disorder Screening Questionnaire; cpRBD, clinical possible rapid eye movement sleep behavior disorder; HAMD-17, 17-item Hamilton Rating Scale for Depression; SCOPA-AUT, the Scale for Outcomes in PD autonomic dysfunction; MMSE, Mini-mental State Examination; NA, not applicable. *p < 0.05; **p < 0.01.

### Comparison of Relative Expression of miR-7, miR-153, and miR-223 in Plasma Between Patients With Parkinson’s Disease and Normal Controls

**FIGURE 1:**
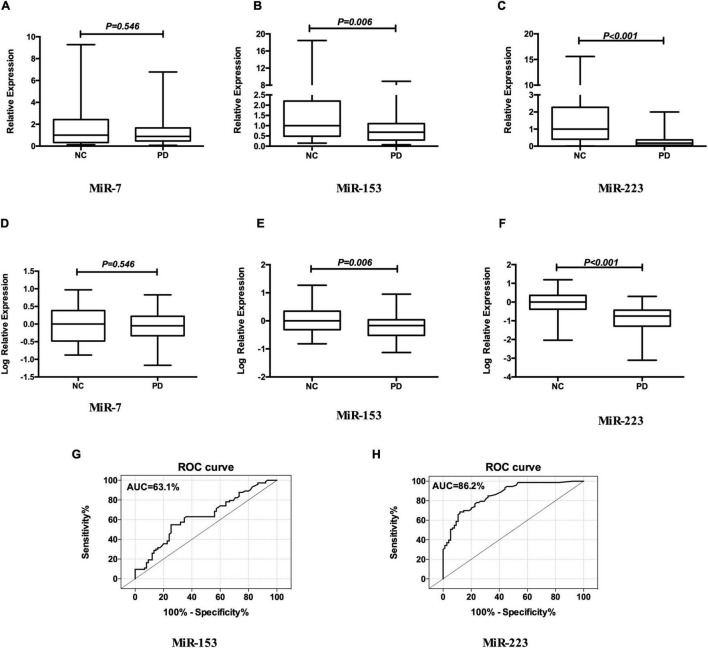
MiR-7, MiR-153, and miR-223 levels in the plasma of patients with PD relative to NCs. Mean expression levels of miR-7 **(A)**, miR-153 **(B)**, and miR-223 **(C)** were determined by real-time quantitative polymerase chain reaction and analyzed using the delta-delta cycle threshold value (2^– Δ^ ^Δ^ *^Ct^*) method. Data were normalized *via* log transformation and reported for miR-7 **(D)**, miR-153 **(E)**, and miR-223 **(F)**. Midline in box and whisker plots depicts the median, with upper and lower limits representing maximum and minimum values, respectively. ROC curves were estimated for miR-153 **(G)** and miR-223 **(H)**, with AUC indicated. *n* = 73 and 75 for NCs and PD groups, respectively. AUC, area under the curve; NCs, normal controls; miR, microRNA; PD, Parkinson’s disease; ROC, receiver operating characteristic.

As shown in Figure 1, the relative expression levels of miR-153 and miR-223 in the plasma of the patients with PD were significantly lower than that of the NCs both in the raw and the log-transformed value. Mean miR-153 concentrations for the patients with PD was 0.68 relative to NCs (*z* = −2.757, *p* = 0.006; Figure 1B). The log-transformed expression levels of miR-153 was significantly different in patients’ with PD with plasma when compared with NCs (*p* = 0.006; Figure 1E). Mean miR-223 concentrations for the patients with PD were 0.18 relative to NCs (*z* = −7.630, *p* = 0.000; Figure 1C). The log-transformed expression levels of miR-223 were significantly different in PD patients’ plasma when compared with NCs (*p* = 0.000; Figure 1F). While the difference in miR-7 concentration and log-transformed expression level between patients with PD and NCs was not statistically significant (0.89 vs. 1.00, *z* = −0.604, *p* = 0.546; Figures 1A,D). MiR-153 distinguished patients with PD from NCs with 54.8% sensitivity and 74.7% specificity (AUC = 0.631, cut-off value 0.85; Figure 1G), whereas, miR-223 distinguished patients with PD from NCs with 68.5% sensitivity and 88.0% specificity (AUC = 0.862, cut-off value 0.63; Figure 1H).

### Relationship Between miR-153, miR-223, and Clinical Phenotype in Parkinson’s Disease

**FIGURE 2:**
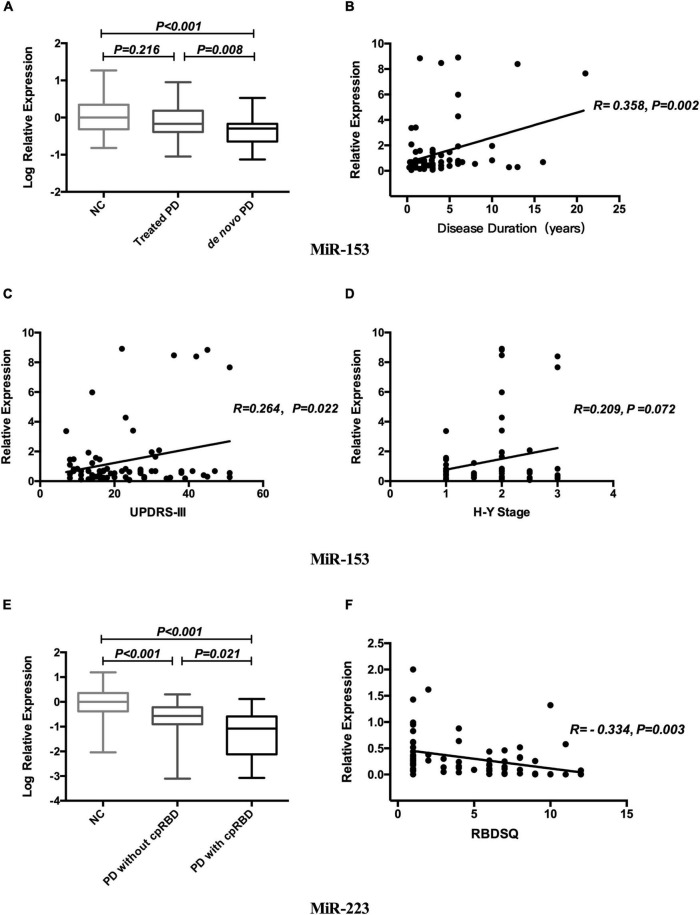
Relationship between miR-153, miR-223, and clinical phenotype in PD. Expression levels of miR-153 in NCs treated PD and *de novo* PD groups **(A)**. Correlation analysis between miR-153 and disease duration **(B)**. Correlation analysis between miR-153 and UPDRS-III **(C)**. Correlation analysis between miR-153 and H&Y stage **(D)**. Expression levels of miR-223 in NCs, PD with cpRBD, and PD without cpRBD groups **(E)**. Correlation analysis between miR-223 and RBDSQ **(F)**. NCs, normal controls; miR, microRNA; PD, Parkinson’s disease; UPDRS-III, Unified Parkinson’s Disease Rating Scale part III; H&Y stage, Hoehn and Yahr stage; cpRBD, clinical possible rapid eye movement sleep behavior disorder; RBDSQ, rapid eye movement sleep behavior disorder screening questionnaire.

Both miR-153 and miR-223 expression levels were not related to gender or age. Log-transformed miR-153 expression level in *de novo* PD was lower than that in the treated patients and in NCs (*p* = 0.008; *p* < 0.001; respectively; Figure 2A). Mean miR-153 concentrations for *de novo* PD are 0.51 and for treated PD is 0.68 relative to NCs. MiR-153 expression level increased gradually with disease duration (*r* = 0.358, *p* = 0.002; Figure 2B) in PD.

Concerning motor symptoms, we found that the miR-153 expression level was positively associated with UPDRS-III scores (*r* = 0.264, *p* = 0.022; Figure 2C) and was marginal correlated to the H&Y stage (*r* = 0.209, *p* = 0.072; Figure 2D).

Regarding non-motor symptoms, log-transformed plasma miR-223 expression level was decreased in patients with cpRBD compared with those without cpRBD and NCs (*p* = 0.021; *p* < 0.001; respectively; Figure 2E), Mean miR-223 concentrations for patients with cpRBD is 0.08 and for those without cpRBD is 0.27 relative to NCs and its level was negatively associated with RBDSQ score (*r* = -0.334, *p* = 0.003; Figure 2F). The mean MOCA-BC score for the patients with PD with cpRBD patients was lower than those without cpRBD (21.00 vs. 24.00, *p* = 0.004).

Multiple linear regression analysis revealed that disease duration (*p* = 0.049) was the only independent associated factor of miR-153 level; whereas, only RBDSQ (*p* = 0.009) was related to miR-223 level in patients with PD.

## Discussion

As a common molecular biomarker for disease diagnosis, plasma miRNAs receive great attention in the research of PD. This is the first study that comprehensively analyzed the expression levels of three SNCA-related miRNAs: miR-7, miR-153, and miR-223 in plasma and explored their diagnostic value in patients with PD *via* serial scales. We found that (1) reduced expression levels of miR-153 and miR-223 in PD relative to healthy controls; (2) miR-153 level correlated with disease duration; whereas, the miR-223 level was related to RBD in patients with PD.

Results of this study showed that the plasma levels of miR-153 and miR-223 were decreased in PD, and they distinguished PD from NC with 63.1 and 86.2% accuracy, respectively, indicating these two miRNAs as potential diagnostic biomarkers of idiopathic PD. Previous studies on miR-153 and miR-223 expression levels in PD vs. NC in different biofluids are summarized in Table 2; Vallelunga et al. (2014),Gui et al. (2015), Zhang et al. (2017), Mancuso et al. (2019), Cressatti et al. (2020). Different specimen sources and sample sizes may contribute to the inconsistency of the results. Our finding is consistent with the results from human saliva. Cressatti et al. (2020) also found miR-153 and miR-223 levels were significantly decreased in the saliva of human patients with PD in comparison with non-neurological controls. It is well known that miR-153 and miR-223 could induce downregulation of SNCA expression by binding the 3’ UTR of SNCA. The reduced expression level of miR-153 and miR-223 in patients’ plasma or saliva indirectly suggests that there may be abnormal expression levels of SNCA in patients with PD.

**TABLE 2:** Serial studies on miR-153 and miR-223 expression levels in PD vs. NC.

Study	Countries	Sample source	Sample size (PD vs. NC)	Expression (PD vs. NC)	*p*-value
**miR-153**					
Gui et al. (2015)	China	Human CSF	78 vs. 35	Up	0.005
Zhang et al. (2017)	China	Human plasma	46 vs. 49	-	0.597
Cressatti et al. (2020)	Canada	Human saliva	83 vs. 77	Down (0.55 vs. 1)	0.01
Our study (2021)	China	Human plasma	75 vs. 73	Down (0.68 vs. 1)	0.006
**miR-223**					
Vallelunga et al. (2014)	Italy	Human serum	25 vs. 25	Up	0.07
Mancuso et al. (2019)	Italy	Human serum	28 vs. 40	Up	<0.001
Cressatti et al. (2020)	Canada	Human saliva	83 vs. 77	Down (0.52 vs. 1)	0.02
Our study (2021)	China	Human plasma	75 vs. 73	Down (0.18 vs. 1)	<0.001

*PD, Parkinson’s disease; NC, normal controls; CSF, cerebrospinal fluid.*

A novel finding is that plasma miR-153 level in *de novo* PD was lower than that in treated patients and its level increased gradually with disease duration and was positively associated with UPDRS-III scores. Further multivariate analysis showed that disease duration is the only independent associated factor of miR-153 level in PD, suggesting that plasma miR-153 level may represent disease progression in PD. Regarding the research related to miR-153 and disease progression, Cressatti et al. (2020) showed that the L-dopa dose increased as a function of the H&Y stage in patients with PD. However, log-transformed miR-153 expression levels were not correlated to LEDD (Cressatti et al., 2020). Narasimhan et al. (2014) reported that miR-153 is significantly upregulated in response to a physiologically relevant level of paraquat (PQ) and miR-153 suppresses Nrf2/ARE cascade and its associated cryoprotection during PQ-induced toxicity in dopaminergic neurons. Therefore, it was speculated that miR-153 may serve an anti-oxidative stress effect in PD, which may partly explain the responsive increase of its level as the disease progression.

Another interesting finding is that plasma miR-223 level was decreased in patients with PD with cpRBD compared with those without cpRBD, and its level was negatively associated with RBDSQ score, indicating that miR-223 is associated with RBD subtype in patients with PD. Numerous recent studies have suggested that patients with PD and RBD have a more diffuse SNCA neuropathology (Postuma et al., 2015a; Leclair-Visonneau et al., 2017). Leclair-Visonneau et al. (2017) showed that enteric phosphorylated SNCA histopathology was more frequent in the subgroup of PD patients with RBD compared with those without RBD. PD patients with RBD have a greater frequency of SNCA pathology in the enteric nervous system (Leclair-Visonneau et al., 2017). Postuma et al. (2015a) found that PD with RBD had increased SNCA deposition in all brain regions examined, with nine of ten regions significantly different. RBD symptoms among patients with PD may have a greater density and range of SNCA pathology on autopsy (Postuma et al., 2015a). Many recent studies have shown that RBD in PD marks a subtype of the disease characterized by an increased risk of cognitive dysfunction and dementia (Dauvilliers et al., 2018). In our PD patients with RBD, the expression level of miR-223 and mean MoCA-BC score was lower than patients without RBD; these data confirm previous results obtained in patients with AD and MCI, showing a significant downregulation of miR-223 in the blood (Mancuso et al., 2019). Moreover, miR-223 induced downregulation of SNCA expression by binding the 3’ untranslated region of SNCA, thus may causing more abnormal deposition of SNCA in patients with PD with RBD.

It should be noted that miR-7 is enriched in the brain and has been found to inhibit the expression of SNCA, there have been a few studies on miR-7 as a biomarker for PD diagnosis in recent years (Zhou et al., 2016; Li et al., 2019; Starhof et al., 2019; Cressatti et al., 2020; Ravanidis et al., 2020). However, the results were inconsistent (Supplementary Table 1). Different sources of tissue and biofluid, sample size, and quality control may partly explain the inconsistency. Also, the connection between the brain and the peripheral bioliquid is complex. Above all, we speculate that miR-7 cannot distinguish PD from NC in some sources of biofluid, especially in plasma and saliva, and may not be used as a diagnostic biomarker of idiopathic PD.

Some limitations should be mentioned in this study. The recruited subjects came from one center, and the sample size is relatively limited; we only compared PD with the NC group and did not enroll the disease control group such as patients with essential tremor or multiple system atrophy. In addition, we did not detect the expression levels of these miRNAs in genetic patients with PD. It is not known whether these miRNAs differ between genetic and idiopathic PD or not. The diagnosis of RBD was based on a subjective questionnaire, not on objective polysomnography (PSG). Blood is easier to obtain than CSF and saliva in the clinical practice. The important finding from this study is that plasma miR-153 and miR-223 levels may serve as useful, non-invasive, and relatively inexpensive diagnostic biomarkers of idiopathic PD. A multi-center cohort study with consistent protocols is warranted in the future to ascertain their correlations with SNCA levels and the diagnostic value on disease development and progression. Moreover, whether plasma miR-223 level is decreased in idiopathic RBD and whether its level could predict phenoconversion from idiopathic RBD to PD merits further investigation.

## Data Availability Statement

The raw data supporting the conclusions of this article will be made available by the authors, without undue reservation.

## Ethics Statement

The studies involving human participants were reviewed and approved by the Medical Ethics Committee of Shanghai Ninth People’s Hospital, Shanghai Jiao Tong University School of Medicine, Shanghai, China (2017-303-T223). The patients/participants provided their written informed consent to participate in this study.

## Author Contributions

LW, QX, MZ, and WC: conceptualization. LW, QX, MZ, and YC: methodology. LW, QX, CJ, YJ, and YL: validation. LW and QX: formal analysis, writing – original draft preparation, and visualization. LW, QX, MZ, YC, CJ, YJ, YL, QH, LZ, and YD: investigation. LW, QH, LZ, YD, JL, and WC: resources. JL and WC: data curation and project administration. QH, LZ, YD, JL, and WC: writing – review and editing. MZ, YC, and WC: supervision. LW and WC: funding acquisition. All authors agreed to the published version of the manuscript.

## Conflict of Interest

The authors declare that the research was conducted in the absence of any commercial or financial relationships that could be construed as a potential conflict of interest.

## Publisher’s Note

All claims expressed in this article are solely those of the authors and do not necessarily represent those of their affiliated organizations, or those of the publisher, the editors and the reviewers. Any product that may be evaluated in this article, or claim that may be made by its manufacturer, is not guaranteed or endorsed by the publisher.

## Footnotes

1
http://mirwalk.umm.uni-heidelberg.de
