# Supplementary material for: Plasma miR-153 and miR-223 Levels as Potential Biomarkers in Parkinson’s Disease
Source: Front Neurosci. 2022 May 17;16:865139. doi: 10.3389/fnins.2022.865139 (PMC9152100; doi:10.3389/fnins.2022.865139)
Supplement: Supplementary file 1 [file Table_1.docx]

**Supplementary Table 1.** Serial studies on miR-7 expression level in PD versus NC.

| **Study** | | **nationality** | **Source** | | | **Sample size**  **(PD vs NC)** | | **Expression**  **(PD vs NC)** | | ***P* value** |
| --- | --- | --- | --- | --- | --- | --- | --- | --- | --- | --- |
| Zhou *et al* (2016) | | China | Human serum | | | 12 vs 12 | | Down (0.7 vs 1) | | <0.05 |
| Ravanidis *et al* (2020) | | Greece | Human plasma | | | 99 vs 101 | | Up (1.28 vs 1) | | 0.038 |
| Li *et al* (2019) | | China | Rat blood | | | 50 vs 50 | | Down | | <0.05 |
| Li *et al* (2019) | China | | | Rat SNpc | 50 vs 50 | | Up | | <0.05 | |
| Starhof *et al* (2019) | | Denmark | Human CSF | | | 118 vs 118 | | Up | | <0.05 |
| Cressatti *et al* (2020) | | Canada | Human saliva | | | 84 vs 83 | | down | | 0.1 |
| Our study | | China | Hunan plasma | | | 76 vs 73 | | Down (0.89 vs 1) | | 0.546 |

Abbreviations: PD, Parkinson’s disease; NC, normal controls; SNpc, substantia nigra pars compacta;
